# Validation of a Targeted LC–MS/MS Method for Cereulide and Application in Food and Faeces

**DOI:** 10.3390/toxins16010013

**Published:** 2023-12-27

**Authors:** Julien Masquelier, Céline Segers, Bram Jacobs, Tom Van Nieuwenhuysen, Laurence Delbrassinne, Els Van Hoeck

**Affiliations:** 1 Organic Contaminants and Additives, Sciensano, Scientific Institute of Public Health, 1050 Brussels, Belgium; 2 Food Pathogens, Sciensano, Scientific Institute of Public Health, 1050 Brussels, Belgium

**Keywords:** cereulide, LC-MS/MS, validation, food, faeces, bacterial toxin

## Abstract

Cereulide is an emetic toxin produced by some strains of *Bacillus cereus*. This bacterial toxin, a cyclic 1.2 kDa dodecadepsipeptide, is stable to heat and acids and causes nausea and vomiting when ingested via contaminated food. This work aimed to develop and validate a targeted analytical method applying liquid chromatography-tandem mass spectrometry (LC–MS/MS) to quantify this toxin in food and human faeces. Samples were extracted with acetonitrile in the presence of ^13^C_6_-cereulide, a labelled internal standard, and purified by centrifugation and filtration. The limits of quantification were 0.5 and 0.3 µg kg^−1^ for food and faeces, respectively. The linearity of the method was very good, with calculated R^2^ values above 0.995. The mean recovery of the method was within the acceptable range of 70.0%–120.0%, the repeatability was not higher than 7.3%, and the highest intra-laboratory reproducibility was 8.9%. The estimated range for the expanded measurement uncertainty was between 5.1% and 18.0%. The LC–MS/MS method was used to analyse one food sample (rice) from a Belgian foodborne outbreak and five faecal samples from patients with clinical symptoms after consumption of the contaminated rice. The levels of cereulide were 12.22 µg g^−1^ for food and between 6.32 and 773.37 ng g^−1^ for faecal samples.

## 1. Introduction

*Bacillus (B.) cereus sensu lato (s.l.)* is a Gram-positive, spore-forming bacteria that poses a significant safety risk in the food industry, with the associated potential production of toxins [1]. According to the European Food Safety Authority (EFSA) annual report, more than 10% of food poisoning outbreaks caused by bacterial toxins are attributed to *B. cereus*, which is more than for *Clostridium* or *Staphylococcus* toxins [2]. With 87 registered outbreaks in the EU in 2021, *B. cereus* is well known for causing foodborne outbreaks due to ingesting foods contaminated by bacteria, toxins, or both. Moreover, the incidence of food poisoning caused by *B. cereus* is certainly underestimated since symptoms are not specific and self-limiting, and reporting procedures may vary between countries [3]. *B. cereus* spores are typically found in soil and can, therefore, contaminate almost all raw materials and unprocessed food. They are responsible for two types of gastrointestinal diseases: emetic or diarrheal syndromes [4]. The diarrheal form of food poisoning is mainly characterised by diarrhoea and abdominal cramps, similar to food poisoning by *Clostridium perfringens* [5]. The enterotoxins known to be involved in this syndrome are hemolysin BL (Hbl), nonhemolytic enterotoxin (Nhe), and cytotoxin K (CytK) [6]. Diarrheic symptoms occur after approximately 10–16 h, and the enterotoxins are produced by viable bacteria inside the human intestine [7]. The emetic form of food poisoning is mainly characterised by nausea, vomiting, and sometimes abdominal cramps combined with diarrhea. Sometimes, fatal emetic cases have been reported. The emetic symptoms, similar to those of *Staphylococcus aureus* intoxication, may appear from 1 to 5 h following ingestion of contaminated food and can continue for approximately 24 h [8]. Due to its ubiquitous nature, *B. cereus* can be found in a wide variety of foods, and the emetic syndrome is due to the ingestion of a preformed toxin, cereulide, mainly produced by *B. cereus* in rice, pasta, potato-based meals, or dairy products [9,10]. Even if *B. cereus*-associated gastrointestinal and emetic diseases are often associated with mild or limited symptoms and are self-limiting with a short duration, severe and fatal outbreaks were also reported in some cases [11,12,13,14]. Moreover, the preformed emetic toxin, cereulide, can also be detected directly in food in such outbreaks. Indeed, low levels of cereulide in food (from 5 to 10 ng g^−1^) have already been reported as illness-inducing doses, while higher levels (µg range) were also reported with various symptoms [12,15,16,17,18].

Cereulide was first described by Agata et al. in 1994 [19]. More recently, other isoforms of cereulide have also been described as showing variabilities in cytotoxicity: isocereulide A shows around 8–10-fold higher cytotoxicity compared to cereulide, while isocereulide B is almost non-toxic [20]. Cereulide is a ring-structured dodecadepsipeptide with a molecular mass of 1.2 kDa, containing three repetitions of four amino acids: D-Oxy-Leu—D-Ala—L-Oxy-Val—L-Val [21]. This small peptide is synthesised by non-ribosomal cereulide synthetase machinery encoded in the ces gene cluster [22] and is chemically very stable and highly resistant to heat (>120 °C during 90 min), acid, and the proteolytic activity of pepsin and trypsin [23,24]. Only highly alkaline pHs can reach inactivation. Consequently, once present in the food chain, cereulide is very difficult to eradicate. Moreover, *B. cereus* spores can survive after thermal treatments, and germinated vegetative cells can grow and produce toxins under favourable conditions [25]. Based on the chemical structure, cereulide is comparable to valinomycin, and both are potassium ion-selective ionophores [26]. Subsequently, it can cause a potassium-dependent drop in the transmembrane potential of mitochondria with the expulsion of H^+^ and stimulation of mitochondrial respiration [27]. However, the precise mechanism of the emetic effect of cereulide remains to be decrypted [28].

The classical microbiological methods for detection of *B. cereus* show bacterial contamination but do not offer any information on the presence or potential concentration of the emetic toxin cereulide that may be produced. Therefore, it is very important to quantify cereulide in food samples, particularly in cases of emetic outbreaks. Typical toxin detection methods depend on enzyme-linked immunosorbent assays (ELISA), but the development of an antibody against cereulide is still challenging. A semi-quantitative system to detect cereulide via a computer-assisted sperm analysis system has already been reported [29], as well as other non-specific bioassays based on Hep-2 cells [30,31], but chemical methods, like LC–MS/MS or Matrix-Assisted Laser Desorption Ionisation–Time of Flight (MALDI-TOF) MS, are now gold standards for its quantification [32,33,34,35,36,37,38]. Chemical methods were used to establish an ISO standard method based on stable isotope MS-based dilution [39], using commercially available synthetic cereulide as an internal standard instead of related compounds such as valinomycin [40,41], allowing precise absolute quantification. Using the original standard effectively decreases matrix effects and interferences in the electrospray ionisation step that could appear when using surrogate standards [42]. ISO 18465:2017 can be applied to analyse the toxin in products intended for human consumption [43].

Numerous analytical approaches for the analysis of cereulide in food are currently well-described and available. However, a similar method covering a wide range of food matrices as well as human clinical samples is an added value to assess potential human consumption and related effects. More particularly, the analytical methods describing cereulide quantification in human samples are very limited in the literature. Even if cereulide was detected in 2010 in human clinical samples (stool, urine, gastric fluid, and blood serum) of outbreak patients by Hep-2 Assay [44], none of the existing methods combine the quantification of cereulide in leftover food and faeces from patients. This study aimed to develop and perform the validation of straightforward and sensitive analytical LC–MS/MS methods in both food and human samples in support of an additional collection of quantitative and occurrence data associated with the intake of food contaminated with cereulide. The method should be able to analyse a broad range of matrices, including lasagna, hotdog sausage, baby food, and rice. Furthermore, it should also be applicable to human samples (faeces), which is a real novelty as there is a real lack of studies on the analysis of cereulide in human samples. This validated method in faeces in combination with food analysis could certainly be useful for further characterisation of *B. cereus* foodborne outbreaks.

## 2. Results and Discussion

### 2.1. LC–MS/MS Use and Optimisation of LC–MS/MS Parameters

Using Liquid Chromatography-Mass Spectrometry (LC–MS) for cereulide detection offers several distinct advantages, making it the preferred analysis method. The rapidity of the method is a huge advantage, as this method could be performed within a couple of hours. High sensitivity allows for detecting cereulide at very low concentrations, which is crucial when dealing with potent toxins like cereulide that can be harmful even in trace amounts. The selectivity of mass spectrometry provides high selectivity by measuring the mass-to-charge ratio of ions. This enables accurate identification and differentiation of cereulide from other compounds in complex food or human matrices. LC–MS can also provide structural information about cereulide molecules. Through fragmentation patterns observed during mass spectrometry analysis, the chemical structure of cereulide can be confirmed, ensuring accurate identification. LC–MS also allows for accurate quantification of cereulide levels in samples. Its linear response over a wide concentration range ensures reliable quantification even at low levels. Food and human samples often contain complex matrices with various interfering compounds. The chromatographic separation capabilities of liquid chromatography resolve interferences and enhance the accuracy of cereulide quantification. Furthermore, this technique can be applied to various samples, including solid, liquid, and semi-solid samples. This versatility allows for the analysis of various matrices, from food products to human samples. Regulatory agencies have recognised and accepted LC–MS methods for cereulide detection, making them suitable for compliance with food safety and quality standards. Modern LC–MS systems also offer rapid analysis times, making it possible to process many samples in a relatively short period of time. The benefits of LC–MS in terms of rapidity, sensitivity, selectivity, and versatility make it a powerful tool for accurate cereulide detection in various matrices.

For the optimisation of LC–MS/MS parameters, an acidic aqueous mobile phase (10 mM ammonium formate (HCOONH_4_) with 0.1% formic acid (HCOOH)) in combination with acetonitrile (ACN) with 0.1% HCOOH as an organic phase was used. Due to the addition of HCOONH_4_, cereulide and its internal standard were always detected as [M + NH4]⁺ adduct.

Considering the MS part of the method, data acquisition were performed in multiple reaction monitoring (MRM) mode to improve selectivity and sensitivity. MRM parameters were adjusted by flow injection analysis in the ESI-positive mode of individual standards (cereulide and ^13^C_6_-cereulide), followed by collision-induced dissociation experiments to identify the most intense product ions. It should be emphasised that the injection of extracts (food and faeces) into the column exhibited parameters comparable with those described in ISO 18465:2017 [43]. Table 1 provides the optimised MRM transitions, cone voltages, and collision energies for each transition and analyte.

**Figure 1:**
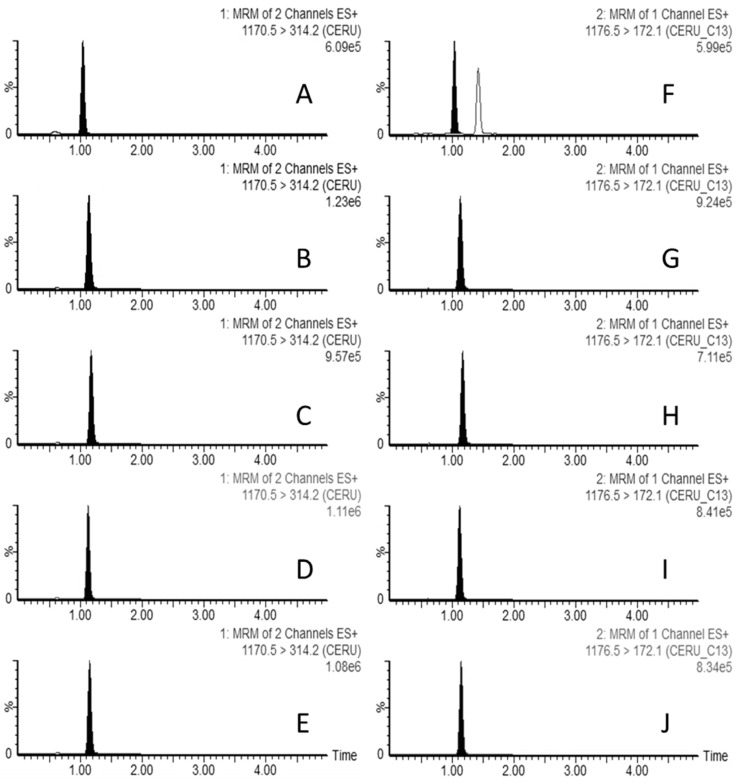
LC–MS/MS multiple reaction monitoring (MRM) chromatograms of a single injection of a cereulide fortified in faeces (**A**), at a concentration of 3 ng g^−1^, rice (**B**), baby food (**C**), hotdog sausage (**D**), and lasagna (**E**), at a concentration of 5 ng g^−1^ and ^13^C_6_-cereulide in faeces (**F**), rice (**G**), baby food (**H**), hotdog sausage (**I**), and lasagna (**J**), at a concentration of 1.7 ng g^−1^. The most prevalent transition is presented for each matrix. The vertical axes represent relative peak intensity (normalised to 100%), while the horizontal axes indicate retention time (in min). The chromatographic conditions used are detailed in Section 4.

To separate the compounds of interest using LC, the suitability of the UHPLC column with Acquity BEH C18 stationary phase, in combination with the acidic mobile phases consisting of ACN with 0.1% HCOOH and the aqueous mobile phase with 10 mM HCOONH_4_ and 0.1% HCOOH, was demonstrated, and good peak shapes were obtained in food and faecal samples using a gradient elution (see Section 4) (Figure 1).

### 2.2. Optimisation of Sample Preparation

A simple ACN extraction procedure described in ISO 18465:2017 was tried for the preparation of food samples [43]. The method demonstrated favourable sensitivity and recovery across various food matrices, such as lasagna, hotdog sausage, baby food, and rice. Because of the complexity of the faecal matrix, the application of a clean-up step in the faecal sample preparation procedure was tested to reduce matrix interference and increase sensitivity. To clean up the extract, solid-phase extraction (SPE) was chosen, and two SPE types, the Strata-X cartridge with a polymeric cation exchange sorbent and the Bond Elut C18 Solid cartridge with a broad retention spectrum among bonded silica sorbents, were tested. Furthermore, several extraction solvents were also tried in combination with these two SPE models. ACN, methanol, and ethanol were used to evaluate the method’s recovery. Unfortunately, these SPE clean-up steps were not found suitable for the purification of faecal samples regarding recovery after purification. In addition, because freeze-drying of faecal samples has emerged as a valuable tool for metabolome research [45,46,47], a freeze-drying step was also tested for this matrix. Even if results were good for higher fortified concentrations, the recoveries obtained for low fortified concentrations (e.g., 0.3 ng g^−1^) were unsuitable (above 120%). Therefore, a simple ACN extraction gave the best recovery results without the SPE purification step on the fresh matrix. Consequently, the application of the ISO 18465:2017 protocol [43] to faecal samples required an adjustment of the volumes used (see Section 4).

For both food and faecal samples, a stable isotope labelled internal standard of cereulide, ^13^C_6_-cereulide, was used to compensate for possible losses during sample preparation and matrix effects, hence optimising the quantification. Using a ^13^C_6_-cereulide internal standard gives better ion suppression correction, accuracy, and precision than using similar compounds [48].

### 2.3. Method Validation

#### 2.3.1. Selectivity and Specificity

For cereulide and ^13^C_6_-cereulide quantitative and qualitative transitions, no interferences were detected in the analysis of blank extracts from blank lasagna, hotdog sausage, baby food, and rice. However, for faecal samples, while the analysis of blank extract was clean for cereulide transitions, an interfering compound was present (Figure 1F) at the ion transition 1176.5 > 172.1 (^13^C_6_-cereulide quantitative transition). Nevertheless, both peaks were well resolved, and there was no identification of any interfering compound with a signal-to-noise ratio greater than three at the retention time of the target compound. Furthermore, all samples fortified with cereulide satisfied the retention time and ion ratio criteria (Commission Decision 2021/808/EC).

#### 2.3.2. Matrix Effect and Linearity

For the analysis of food samples, and based on the protocol described in ISO 18465:2017, calibration curves were made in ACN. For faecal samples, after an evaluation of the slopes of calibration curves prepared in solvent and in blank matrix extract, the statistical *t*-test (at 95% confidence) illustrated that there is no significant difference between the slopes in the solvent and in the matrix and, therefore, no presence of matrix effects (Table 2). Consequently, the calibration curves were established extemporaneously in the solvent (ACN) for food and faecal samples.

The linearity of the calibration curves of cereulide in solvent was evaluated with Mandle’s fitting test. The linear regression model was selected as the test value was lower than the *F*_crit_ value (Table 2). Moreover, coefficients of determination above 0.995 were always obtained during the analyses. The linear regression was assessed using a calibration range between 0.01 ng mL^−1^ and 8.3 ng mL^−1^, equivalent to 0.1 µg kg^−1^ and 83.3 µg kg^−1^ in matrix (food and faeces).

#### 2.3.3. Limits of Detection and Limits of Quantification

The LOD and LOQ were determined as the lowest concentration in the calibration curve or the lowest level meeting the validation criteria, respectively, which, in our opinion, ensures the method quality. The LOD was 0.1 µg kg^−1^ for both food and faecal samples, corresponding to the lowest concentration in the calibration curve. The LOQ was set at 0.3 and 0.5 µg kg^−1^ for faeces and food, respectively, as these were the lowest validated concentrations. The average signal-to-noise values for the LOD were always above three, while the values for the LOQ were consistently higher than ten. The limits of detection and/or quantification of the validated method were equivalent or better compared to several previously described methods in food [35,49,50,51].

#### 2.3.4. Apparent Recovery, Repeatability, Reproducibility, and Measurement Uncertainty

The apparent recovery values for every fortified sample consistently exceeded 70%. This was independent of the concentration level and matrix tested. The mean values (Table 3) fell within the acceptable range of 70% to 120%, and the lowest observed apparent recovery was 92.1% for cereulide at the intermediate level in food (i.e., 5.0 μg kg^−1^). At the lowest concentration levels (0.5 and 0.3 µg kg^−1^ for food and faeces, respectively), apparent recoveries were 109.2% and 100.9% for food and faecal samples, respectively. The highest apparent recovery was 111.0% in faeces at an intermediate concentration level (2.9 µg kg^−1^).

Furthermore, Horwitz ratios determined the maximum acceptable repeatability and reproducibility in each matrix. The repeatability and reproducibility calculated with the results from the validation were below these maximum values. The method’s repeatability was lower than 7.3%, and the highest intra-laboratory reproducibility was 8.9% in faeces at an intermediate concentration level (Table 3). The highest measurement uncertainty (MU) was 18% in faeces.

In general, the criteria for apparent recovery, repeatability, intra-laboratory reproducibility, and measurement uncertainty were satisfied at every concentration level of cereulide and for all the tested matrices during the validation process (including lasagna, hotdog sausage, baby food, rice, and faeces).

In brief, an LC–MS/MS method for quantification of *Bacillus cereus* emetic toxin, cereulide, was successfully validated in food (lasagna, hotdog sausage, baby food, and rice) and faeces.

#### 2.3.5. Method Application to Food and Human Samples

In 2022, in Belgium, a dish of cooked rice was served among other dishes during a party. Several people became sick after eating this rice. The Belgian National Reference Laboratory for Foodborne Outbreaks also received faeces from five patients with clinical signs after ingesting the contaminated rice. The faecal samples were collected between one and five days after the onset of symptoms. The developed and validated LC–MS/MS method was applied to analyse one food sample (rice) and five faecal samples from patients with clinical symptoms after the consumption of contaminated rice. The rice sample was highly contaminated, with a quantification of cereulide at 12.22 µg g^−1^. This level of contamination (range of µg g^−1^ for cereulide in food) is usual in foodborne outbreaks. Several other cases were reported in a similar high-level range [12,18,50,52].

**Figure 2:**
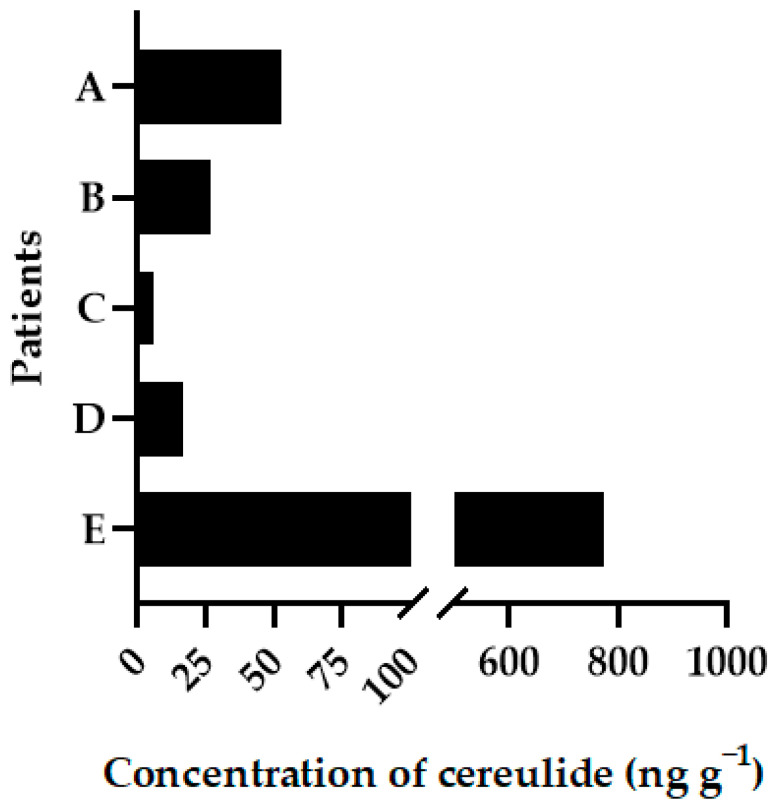
Quantification of cereulide in faeces samples of five patients (A–E) with symptoms after ingestion of contaminated rice.

The samples were analysed following the validated method, and results ranged between 6.32 and 773.37 ng g^−1^ in faeces, confirming the cause of the symptoms due to the ingestion of contaminated meals and the subsequent excretion of the toxin (Figure 2).

These results suggest that the concentrations of cereulide in the human faecal sample after ingestion of contaminated meal are still relatively high (higher than 700 ng g^−1^) and consistently detectable with this validated method when patients show typical clinical signs such as vomiting or diarrhea. Therefore, reliable monitoring of faeces from patients suspected to be intoxicated with cereulide-contaminated food could be helpful in the assessment of intoxication.

The quantity of publications on LC–MS/MS detection methods focusing on food or foodborne outbreak samples is increasing in the literature. Even if there is no official regulation for cereulide levels in food, reaching low levels of LOQ and satisfactory performance is essential to detecting even limited amounts. Nevertheless, there is a lack of methods for the detection of cereulide in human samples (urine, plasma, faeces, etc.). Such detection methods could be helpful to characterise the origin of contamination, given that symptoms are often similar after bacterial toxins have been contaminated.

## 3. Conclusions

In conclusion, this study focused on the development and validation of a sensitive analytical LC–MS/MS method for the quantification of *Bacillus cereus* emetic toxin, cereulide, in various food matrices (lasagna, hotdog sausage, baby food, and rice) and human faecal samples. The method demonstrated several advantages, including high sensitivity, selectivity, and versatility, making it a powerful tool for cereulide detection in complex matrices. The validation process confirmed the method’s robustness, meeting the criteria for apparent recovery, repeatability, reproducibility, and measurement uncertainty across different concentration levels and matrices. The method’s sensitivity was demonstrated by its low limits of detection and quantification, making it suitable for detecting cereulide even at trace levels in food and faeces. The method was successfully applied to analyse a highly contaminated rice sample from a foodborne outbreak and human faecal samples from patients with clinical symptoms after consuming contaminated rice. The results indicated significant cereulide contamination in food and human samples, confirming its role in food poisoning outbreaks and its excretion in detectable amounts in faeces.

Overall, this validated LC–MS/MS method provides a valuable tool for monitoring and assessing cereulide contamination in food and human samples, enhancing our understanding of *Bacillus cereus*-related foodborne illnesses and their impact on public health.

## 4. Materials and Methods

### 4.1. Standards, Reagents, and Consumables

Analytical standards of cereulide and an isotopically labelled internal standard, ^13^C_6_-cereulide, were purchased from Chiralix B.V. (Nijmegen, The Netherlands). Individual stock solutions of standards were bought as acetonitrile solutions with a concentration of 50 µg mL^−1^ and 20 µg mL^−1^ for cereulide and ^13^C_6_-cereulide, respectively. Intermediate solutions were prepared by diluting the stock solutions in ACN. Intermediate and working solutions were made at 250 and 10 ng mL^−1^ for cereulide and 1000, 100, and 10 ng mL^−1^ for ^13^C_6_-cereulide by diluting the individual stock solutions in ACN.

All standard solutions were always stored at −20 °C.

Acetonitrile ULC-MS grade (ACN), formic acid 99% LC–MS grade (HCOOH), and ammonium formate ULC-MS grade (HCOONH_4_) were purchased from Biosolve (Valkenswaard, The Netherlands). Water (H_2_O) was purified using a Milli-Q system (Millipore, Overijse, Belgium). The water exhibited a resistivity of 18.2 Ω and a total organic carbon content ranging from 2 to 3 ppm. Two mL syringes for filtration and 50 mL centrifugal tubes were acquired from VWR (Leuven, Belgium), and the 0.2 µm polytetrafluoroethylene (PTFE) syringe filters were purchased from Phenomenex (Utrecht, The Netherlands). Two mL amber glass injection vials were purchased from Waters (Waters, Milford, MA, USA).

### 4.2. Instrumentation and LC–MS/MS Conditions

The liquid chromatography instrument employed for this study was an ACQUITY UPLC H-Class system (Waters, Milford, MA, USA) fitted with a quaternary pump, sampler manager, and column oven. The LC separation of cereulide was obtained on a reversed-phase ACQUITY UPLC BEH C18 column (50 × 2.1 mm, 1.7 μm) (Waters) with a pre-filter (0.22 μm) (Waters, Milford, MA, USA). The temperature of the column was adjusted to 40 °C. The aqueous mobile phase was made up of H_2_O with the inclusion of 10 mM HCOONH_4_ and 0.1% of HCOOH, while the organic mobile phase was ACN with the addition of 0.1% of HCOOH. For the food sample, the elution was conducted with a flow rate of 1 mL min^−1^ employing the subsequent gradient program: 0–1.2 min: 92% B; 1.2–1.8 min: 92–100% B; 1.8–2 min: 100–92% B. The total run time was 2 min, and the injection volume was 10 μL. For the faecal samples, the elution was performed at a flow rate of 1 mL min^−1^ using the following gradient program: 0–1.2 min: 92% B; 1.2–2 min: 92–100% B; 2–4.8 min: 100% B; 4.8–5 min: 92% B. The total run time was 5 min, and the injection volume was 10 μL.

Following the separation by liquid chromatography, cereulide and ^13^C_6_-cereulide were detected with an XEVO TQ-S triple quadrupole mass spectrometer (Waters, Milford, MA, USA) fitted with an electrospray ionisation (ESI) source operated in positive mode. The optimised configurations for the MS instrument were as follows: source temperature 150 °C, desolvation temperature 550 °C, desolvation gas flow 1200 L h^−1^, cone gas flow 150 L h^−1^, collision gas flow 0.17 mL min^−1^ with argon as collision gas. The value of 60 V was set for the source offset and 3 kV for the capillary voltage. The acquisition of MS data were performed in Multiple Reaction Monitoring (MRM) mode. The selected MRM parameters for cereulide quantification are listed in Table 1. The transition displaying the highest signal intensity (quantifier) was employed for quantifying the analyte, while the second most intense ion transition was utilised for identification (qualifier).

### 4.3. Preparation of Calibration Standards and Quality Control Standards

Quality control (QC) measures were established to guarantee the daily method performance and ensure the reliability of analytical results, including the analysis of fortified samples and replicate injections of one of the calibration points—system suitability checks (SSC). For the apparent recovery of the sample, during each analytical run, a blank sample fortified with cereulide standard before extraction is analysed following the same protocol as the unknown samples. The accepted range of the apparent recovery of the QC sample was 70–120%. This sample is used to verify whether the sample preparation step has worked properly. The criteria for the identification of cereulide in samples for analysis were according to Regulation (EU) 2021/808 (Commission Decision 2021/808) and ISO 18465:2017 [43,53]. For SSC, at the beginning of each analytical run, a control with a concentration of 0.83 ng mL^−1^ was injected at least three times to verify that the LC–MS was working correctly, i.e., signal intensity variation for consecutive injections and retention time variation between injections.

### 4.4. Samples

The food sample, a cooked rice dish, was taken by the Belgian Federal Agency for the Safety of the Food Chain following similar complaints from several consumers. This sample was ground and homogenised before the extraction step. The faecal samples were obtained from patients admitted to the hospital following clinical symptoms (including diarrhoea and vomiting) after the consumption of the contaminated rice. Volunteers provided blank samples of human faeces.

### 4.5. Sample Preparation

For the preparation of food samples, the process is based on the ISO 18465:2017 method [43]. Briefly, a solid-liquid extraction (SLE) was applied. 3 ± 0.05 g of sample were weighted in a 50 mL centrifugal tube, fortified with an internal standard solution, and extracted with 30 mL of ACN after 30 min of equilibration. The process is adapted from ISO 18465:2017 for the preparation of faecal samples preparation [43]. An SLE was also applied, but 1 ± 0.02 g of faeces was weighted in a 50 mL centrifugal tube, fortified with an internal standard solution, and extracted with 10 mL of ACN after 30 min of equilibration.

For both sample types, after shaking for one hour and centrifugation for 10 min, the extracts were filtered through a PTFE filter (0.2 µm) fixed on a syringe into an amber glass vial. The extracts were then analysed by LC–MS/MS.

### 4.6. Method Validation

The method validation was assessed in four food matrices (lasagna, hotdog sausage, baby food, and rice) and human faeces.

The following parameters were determined during method validation: selectivity and specificity, linearity, sensitivity (limits of detection (LOD) and quantification (LOQ)), repeatability, intra-laboratory reproducibility, and measurement uncertainty. The method’s selectivity and specificity were assessed by analysing and comparing blank matrix samples (food and faecal samples) and fortified matrix samples. These parameters were evaluated, showing that on the one hand, no compound interferes with or can disrupt the identification and quantification of the cereulide in the blank matrix or injection solvent, and on the other hand, when cereulide is fortified in the sample or injection solvent, the presence of a chromatographic peak is confirmed at the retention time expected for this compound without interference. To assess linearity, standard solutions of cereulide were formulated in the injection solvent (ACN). The linearity was evaluated with Mandel’s test to compare the linear and quadratic regression models. The linear regression model was preferred if the test value was lower than the critical value, *F*_crit_. If the test value was higher than *F*_crit_, the quadratic model was chosen. At least six calibration points in triplicate (on three different days) were injected to assess the linearity. To determine apparent recovery, repeatability, and intra-laboratory reproducibility, the fortified matrix samples were prepared at 0.5, 5, 15, and 80 μg kg^−1^ for food matrices. For faecal samples, the validation was performed at 0.3, 2.9, and 29.2 μg kg^−1^.

For lasagna, hotdog sausage, baby food, and rice, a one-day validation for each matrix was performed with the samples fortified at each concentration level in triplicate. For faeces, the samples were prepared at each concentration level in triplicate, and the experiment was repeated over three different days. The apparent recovery was calculated by taking the ratio between the measured and the fortified concentration and expressing it in percentages (%). Repeatability and intra-laboratory reproducibility were expressed as the relative standard deviation (RSD). The measurement uncertainty was determined by calculating intra-laboratory reproducibility and applying a coverage factor of 2 for 95% confidence. The LOD was determined as the lowest concentration of cereulide with a signal-to-noise ratio (S/N) of at least three, and the lowest point of the calibration curve was selected. At the same time, the LOQ was defined as the lowest concentration of cereulide, which can be quantified by the method and which met the validation criteria.

## Figures and Tables

**Table 1:** Retention time and optimised MRM parameters for the detection of cereulide and ^13^C_6_-cereulide in food and faeces.

Standard	Precursor ion	Cone Voltage	Product Ions (*m*/*z*) * [Collision Energy in eV]
Cereulide	1170.5	60	**314.2** [65]
		60	499.3 [65]
^13^C_6_-cereulide	1176.5	60	**172.1** [90]
		60	315.4 [75]

* The daughter ions highlighted in bold were used as quantifiers, while the second ions were considered qualifiers.

**Table 2:** Evaluation of matrix effects and linearity for the quantification of cereulide in faeces.

Matrix			
	Matrix effect		
	*t*-value	*t*_crit_ (95%)	Matrix effect
Faeces	1.37	2.03	No
	Linearity		
	*t*-value	*F*_crit_	Linear model
Solvent	6.73	7.82	Yes

**Table 3:** Validation data for cereulide in food (lasagna, hotdog sausage, baby food, and rice) and faeces matrices (linear range, apparent recovery, repeatability, intra-laboratory reproducibility, and measurement uncertainty).

Analyte	Linear Range (µg kg^−1^)	Concentration Level (µg kg^−1^)	Apparent Recovery (%)	Repeatability (%)	Reproducibility (%)	Measurement Uncertainty (%)
Food	0.1–83.3	0.5	109.2	5.2	5.2	10.4
		5.0	92.1	1.8	5.4	10.7
		15.0	96.4	3.0	3.7	7.3
		80.0	98.3	2.8	4.2	8.5
Faeces	0.1–83.3	0.3	100.9	4.9	5.3	10.6
		2.9	111.0	7.3	8.9	18.0
		29.2	105.4	1.5	2.5	5.1

## Data Availability

Data are contained within the article.
